# Supplementary material for: Field programmable spin arrays for scalable quantum repeaters
Source: Nat Commun. 2023 Feb 9;14:704. doi: 10.1038/s41467-023-36098-8 (PMC9911411; doi:10.1038/s41467-023-36098-8)
Supplement: Supplementary file 1 — Supplementary Information [file 41467_2023_36098_MOESM1_ESM.pdf]

Supplementary Information for Field Programmable Spin Arrays for Scalable Quantum Repeaters

Hanfeng Wang[1,2], Matthew E. Trusheim[1,3,*] Laura Kim[1,4], Hamza Raniwala[1,2], and Dirk R. Englund[1,2†]

[1] Research Laboratory of Electronics, M.I.T, 50 Vassar Street, Cambridge, MA 02139, USA

[2] Department of Electrical Engineering and Computer Science, M.I.T., Cambridge, MA 02139, USA

[3] U.S. Army Research Laboratory, Sensors and Electron Devices Directorate, Adelphi, MD 20783, USA

[4] Department of Materials Science and Engineering, University of California, Los Angeles, Los Angeles, CA, 90095 USA

I. SUPPLEMENTARY NOTE 1: DEVICE DESIGN CONSIDERATIONS

For the photonic crystal structure design, we considered many photonic crystal structures but we found that the one shown in Fig. 1 is more suitable for the FPSA because of the merits listed below:

(1) There are many types of photonic crystal cavity structures proposed in previous works such as [1]. However, in our proposal, we want many qubits coupled in the same waveguide mode and they can interact via a single waveguide mode. So a slow light waveguide is more suitable in our case.

(2) The fin structure with large dielectric constant can localize the electric field. In the electric field-based FPSA design. The HfO_2 fin structure not only acts as the photonic crystal structure but also acts as electric field concentrators. These HfO_2 E-field concentrators with a high DC refractive index of ~ 23 will decrease the cross-talk and increase the electric field in diamonds. In Supplementary II, we compare the cases with/without fin structures. Based on COMSOL simulations, The field maximum and the spatial refinement are reduced by a factor of 1.7, increasing cross-talk fidelity from 0.66 to 0.92.

(3) For strain-based FPSA, we use piezoelectric material (e.g. AlN) instead of HfO_2 to transfer the strain to the diamond and get an efficient strain tuning on defects in the diamond.

(4) This structure can be easily fabricated by etching AlN/HfO_2 and transferring the diamond waveguide using the pick-and-place process.

II. SUPPLEMENTARY NOTE 2: ELECTRIC FIELD CONFINEMENT BY DIELECTRIC FINS

In the main text, Fig. 2(a) shows the electric field profile of the electric field-based FPSA (eFPSA) which uses fin structure to confine electric field. For a comparison, we show the field profile without dielectric guiding fins in Supplementary Fig. 1. The field maximum and the spatial refinement are both reduced by a factor of 1.7, resulting a cross-talk fidelity of $F = 0.66$.

* mtrush@mit.edu

† englund@mit.edu

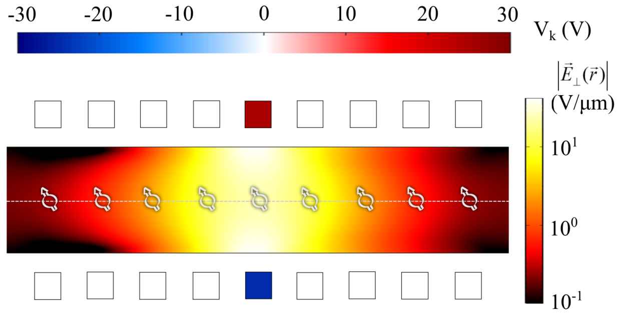

Supplementary Figure 1. Electric field profile without dielectric fins

III. SUPPLEMENTARY NOTE 3: EQUIVALENT CIRCUIT OF FPSA

The FPSA can be modeled as a capacitor C and a parallel resistance R. We neglect the inductance because $\omega L \ll 1/\omega C$ in the FPSA. We consider a circuit shown in Supplementary Fig. 2(a), where a voltage source U and the eFPSA are connected by a transmission line with length $l = l_1 + l_2$. Here l_1 (l_2) is the length of transmission line in low temperature (room temperature) part. The low temperature part contains an FPSA, a series resistance R_w with a parallel wire capacitor $C_w \sim$ fF/μm [2] and a transmission line with length $l_2 \ll c/f \sim 0.15$ m. The room temperature (RT) part contains a transmission line with length $l_1 \gg l_2$ and a voltage source U.

In the following calculation, we omit the wire capacitor C_w because $R_w \ll 1/j\omega C_w$. The impedance of the low-temperature part is:

$$Z_{LT} = Z_0 \frac{Z_C + jZ_0 \tan(\beta l_2)}{Z_0 + jZ_C \tan(\beta l_2)} \approx Z_C \tag{1}$$

where $Z_0 = 50\ \Omega$, $\beta = 2\pi/\lambda$ the wavenumber, and

$$Z_C = \frac{R(1 - j\omega CR)}{1 + \omega^2 C^2 R^2} + R_w \tag{2}$$

Similar to the open circuit, the potential difference across the eFPSA is:

$$U_e = \frac{2Z_C}{Z_C + Z_0} U \sim 2U \tag{3}$$

where U_0 is the source voltage. The voltage on eFPSA is $2U_0$ and the current is almost zero.

The heat load per π-pulse in the low temperature is:

$$J_E = (\frac{(U_e)^2}{R} + |\frac{U_e}{1/j\omega C}|^2 R_w)\frac{1}{4\Omega_R} \tag{4}$$

with

$$U_e = \frac{\Omega_R \Lambda}{d_\perp} \tag{5}$$

then we have:

$$J_E = \frac{1+\omega^2 C^2 R_w R}{R}\frac{\Lambda^2\Omega_R}{4d_\perp^2} \tag{6}$$

Then we consider a circuit shown in Supplementary Fig. 2(b) for magnetic field-based spin control. Here we model the low temperature part as a wire capacitor C_w and a parallel resistance R_w, for the same reason we omit the wire capacitor C_w in the calculation. The heat load per π-pulse in the low temperature part for this circuit is:

$$J_B = \frac{\pi^2}{\mu_0^2\gamma^2} d^2 R_w \Omega_R \tag{7}$$

where d is the distance between wire and NV centers. Here we assume $\Lambda = d$ then we have:

$$\frac{J_E}{J_B} \sim \frac{\mu_0^2\gamma^2}{4\pi^2 d_\perp^2}\frac{1+\omega^2 C^2 R_w R}{R_w R} \tag{8}$$

Similarly, the power dissipation of the strain-based FPSA can be written as:

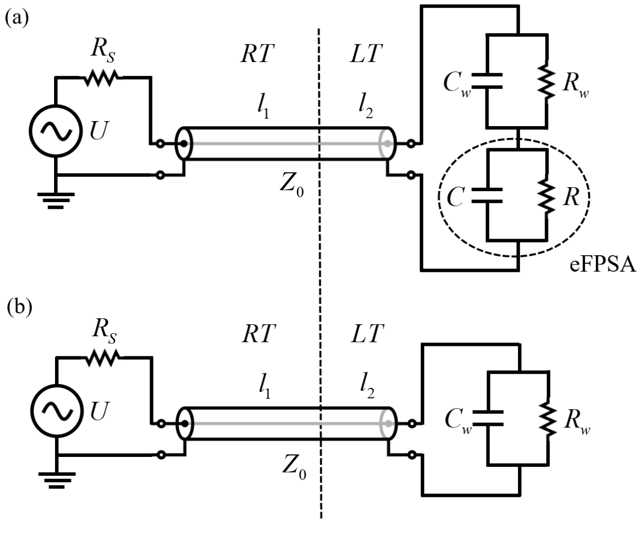

Supplementary Figure 2. (a). Equivalent circuit of eFPSA. Room temperature (RT) part contains a transmission line with length l_1 and a voltage source U. Low temperature (LT) part contains a transmission line with length l_2 and eFPSA. (b). Equivalent circuit for magnetic field-based control.

$$J_S = (\frac{U_e^2}{R} + |\frac{U_e}{1/j\omega C}|^2 R_w)\frac{1}{4\Omega_R} \tag{9}$$

Here

$$\Omega_R = -\frac{\gamma_S\sqrt{\beta^2+\gamma^2}B_\perp}{\lambda_{\mathrm{SO}}} \tag{10}$$

where β and γ are the magnitudes of transverse AC strain fields that couple to the SiV spin, $B_\perp$ is a static transverse magnetic bias field, and λ_{SO} is the spin-orbit coupling strength. The capacitance of the strain-based FPSA is $C = 3.5 \times 10^{-17}$ F. Assuming a transverse bias field of $B_\perp = 0.17$ T as used in prior work [3] with U_e = 0.14 V to reach $\Omega_R = 20$ MHz and a $\sim$ PΩ leakage resistance [4], the heat load per π-pulse in the low temperature part for this circuit is 2.5×10^{-25} J.

As a comparison, the heat load per π-pulse in the low temperature part for microwave control is:

$$J_B = \frac{\pi^2 d^2}{\mu_0^2} R_w \frac{\Omega_R \lambda_{\mathrm{SO}}^2}{\gamma_S^2(\beta^2+\gamma^2)} \tag{11}$$

Here we assume $\beta = -1.29 \times 10^{11}$ Hz based on [5], the heat load per π-pulse in the low temperature part for microwave control is 1.7×10^{-17} J. Then we have $J_S/J_B = 8.9\times10^{-9}$. In the real case, we need to consider the leakage current.

IV. SUPPLEMENTARY NOTE 4: COMPARISON OF FIELD LOCALIZATION BETWEEN MAGNETIC FIELD-BASED SPIN DRIVING AND EFPSA

We compare the localization of electric field and magnetic field based on example geometries to derive the scaling laws. In the magnetic field case we consider the single line, two lines, loop, loop and feed lines shown in Supplementary Fig. 3(a-c). In the electric field case, we consider electrode pair and eFPSA shown in Supplementary Fig. 3(d) and Fig. 1(a). Here we assume the distance between the NV and nearest structure is $a = 250$ nm for all geometries, and normalize the field at $z = 500$ nm for comparison.

We show the calculated field profiles in Supplementary Fig. 3(c), using Biot-Sarvart law for magnetic field and COMSOL simulation for electric field geometries. For a single wire, magnetic field falls slowly with $B \propto 1/r$. Structures with two opposing currents will locally cancel the magnetic field. The magnetic field from these structures (two-lines and loop) falls off as $B \propto 1/r^2$ and $B \propto 1/r^3$. However, the loop may need to be connected by feed lines for transferring current shown in Supplementary Fig. 3(c). Then the scaling then will be limited by $B \propto 1/r^2$. In the electric field case, we consider an electrode pair, the electric field will scale as $E \propto 1/r^3$. For eFPSA, despite the many electrodes connected to the ground to help localize the electric field, the electric field still scales as $E \propto 1/r^3$.

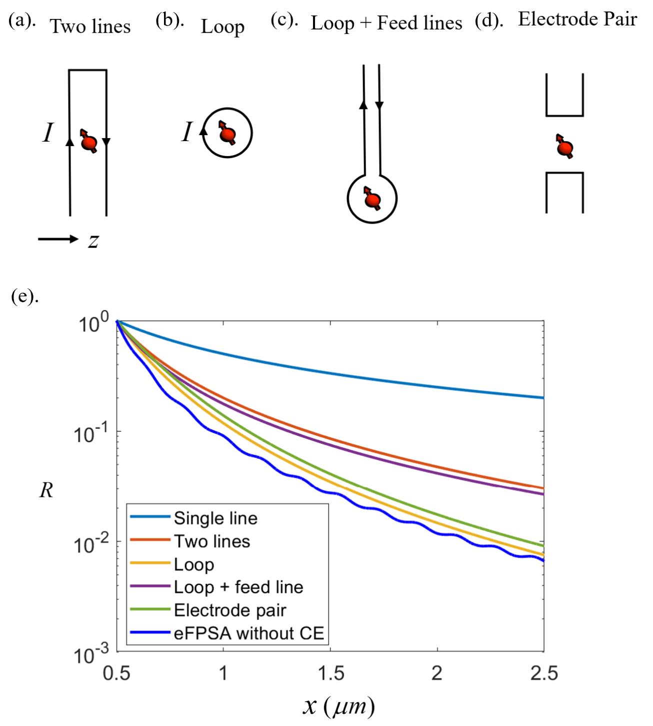

Supplementary Figure 3. Field (a). Two lines structure, where NVs are set between two lines. (b). Loop structure, where NVs are set in the center of the loops. (c). Loop structure with feed lines. (d). Field profile for all structures normalized by the field at 500 nm.

V. SUPPLEMENTARY NOTE 5: ENTANGLEMENT GENERATION WITH SUPERRADIANCE IN A PHOTONIC CRYSTAL WAVEGUIDE

Here we consider the entanglement scheme for an NV pair in a photonic crystal waveguide. We prepare two NVs in $|\Psi\rangle = |11\rangle + |01\rangle + |10\rangle + |00\rangle$ and excite $|0\rangle$ to $|E_y\rangle$, as shown in Supplementary Fig. 4(a) and Supplementary Fig. 4(b). We then treat the spontaneous emission of each component of $|\Psi\rangle = |11\rangle + |E_y 1\rangle + |1E_y\rangle + |E_y E_y\rangle$ individually. The $|11\rangle$ state will remain unchanged under optical emission timescales. The $|E_y 1\rangle$ or $|1E_y\rangle$ states will emit a single photon with spontaneous emission rate $\Gamma_{sp} = 100$ MHz [6] (Supplementary Fig. 4(c)), where we assume a Purcell factor of 10. Due to the superradiance effect in the photonic crystal waveguide, the $|E_y E_y\rangle$ state will first radiatively decay with rate $2\Gamma_{sp}$ to the bright Dicke state $|B\rangle = |E_y 0\rangle + e^{ikL}|0E_y\rangle$, where L is the distance between two NVs and k is the photon wavevector [7]. Then another photon is emitted when the Dicke state decays to the ground state at rate $2\Gamma_{sp}$. The decay rate of $2\Gamma_{sp}$ is the critical difference between independent emission and superradiant emission in the photonic crystal waveguide.

In the quantum entanglement generation scheme, the main infidelity is misheralding. Upon single photon detection, the desired state is $|\Psi_0\rangle = |01\rangle + |10\rangle$ following from the $|E_y 1\rangle + |1E_y\rangle$ state. However, a single photon can also be detected after the spontaneous emission by $|E_y E_y\rangle$ and subsequent loss, in which case the state is $|00\rangle$ and misheralding has occurred. In the single photon scheme [8], the probability of $|E_y E_y\rangle$ is reduced by preparing a superposition state $|\alpha\rangle = \sqrt{\alpha}|0\rangle + \sqrt{1-\alpha}|1\rangle$. By choosing α factor, the fidelity $F = 1 - \alpha$ will be traded-off with entanglement rate $r = 2\alpha p_{det}$. In Barrett-Kok scheme [9, 10], this error is eliminated by flipping the spin and repeating the optical heralding process, where a second photon detection is not possible from the $|11\rangle$ state. This two-step scheme leads to an entanglement rate $r = p_{det}^2/2$.

The change of the lifetime in a slow-light waveguide offers a potential path to decrease the infidelity due to misheralding. Since the emission from the desired state has a longer lifetime, we can differentiate superradiance and standard cases. Specifically, the detection of a single photon at t results in a fidelity:

$$F = \frac{\exp(\Gamma_{sp} t)}{2 + \exp(\Gamma_{sp} t)} \tag{12}$$

However, the probability density for detecting a photon at $t \to t + dt$ is:

$$p(t) = \Gamma_{sp} \exp(-\Gamma_{sp} t) \tag{13}$$

Finally the rate-fidelity trade-off for this process can be written as:

$$p = \frac{1-F}{2F} \tag{14}$$

As shown in Supplementary Fig. 4(e), the photon detected after $t_0 = 53$ ns heralds the entanglement generation with a fidelity $F > 0.99$. The probability to get a photon after $t_0 = 53$ ns is $p = 5 \times 10^{-3}$. We compare the rate using several schemes with $F = 0.99$ in Supplementary Fig. 4(f). Used alone, the time-domain filtering doesn't give an advantage over other schemes. However, timing information can be recorded in conjunction with other schemes. For example, a photon detection at $t > t_0$ already heralds high-fidelity entanglement in a Barrett-Kok scheme, rendering the second heralding step unnecessary for eliminating heralding error. Considering the photon detection after the first step, the combination of Barrett-Kok scheme and superradiance gives the best rate when photon detection efficiency $p_{det} > 3 \times 10^{-2}$.

VI. SUPPLEMENTARY NOTE 6: QUANTUM REPEATER SIMULATION

A. Simulation details

Step 1: Distant entanglement between A(B) and electron spin $|j_e\rangle(|k_e\rangle)$ in the FPSA. Here we choose a heralded single-photon scheme [8], which has previously

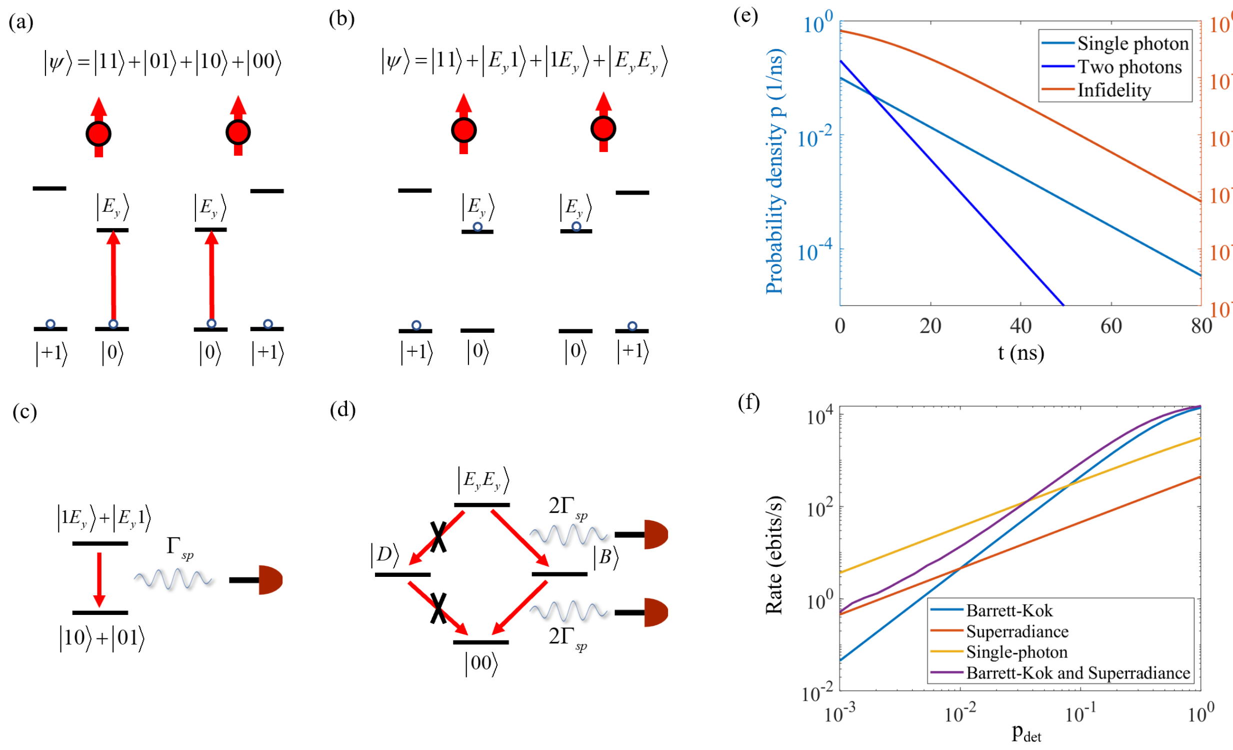

Supplementary Figure 4. Entanglement generation between two NVs. (a). Two NVs are prepared at state $|\Psi\rangle = |11\rangle + |01\rangle + |10\rangle + |00\rangle$. (b). Both NVs are optically excited to $|\Psi\rangle = |11\rangle + |E_y 1\rangle + |1E_y\rangle + |E_y E_y\rangle$. (c). Superradiance spontaneous emission with rate $2\Gamma_{sp}$ when NV is at $|E_y E_y\rangle$. (d). Single photon spontaneous emission with rate Γ_{sp} when NV is in $|1E_y\rangle + |E_y 1\rangle$. (e). Probability for emitting a photon at time t for cases (c) and (d) and the infidelity to distinguish both cases. (f). Entanglement rate for Barrett-Kok scheme, single-photon scheme, superradiance and a combination of superradiance and Barrett-Kok scheme.

been demonstrated for NV centers [8], followed by swapping to the ^{15}N nuclear spin $|j_n\rangle(|k_n\rangle)$. After an NV charge/resonance (CR) state check, each NV will make attempts with time-frequency multiplexing. The successful probability for every qubit is:

$$p_j^s = 2\alpha \exp(-\gamma L) p_d p_c \beta \exp(-t_{wg} N_j)/2 \tag{15}$$

with $j = 1, 2, ..., N/2$ are the qubit indices. where $p_d = 0.83$ ($p_c = 0.33$) is the detection (quantum frequency conversion) efficiency. We conservatively assume a lower $F_P = 10$ ($\beta = 25\%$) to avoid high loss and fabrication sensitivity in the regime of high group index. Here we set $\alpha = 0.01$ to keep the two-photon excitation error of this scheme below 1%. Here we assume a single-side detection for the first step, where we use the left (right) detector to generate distant entanglement with Alice (Bob) and neglect the photon to right (left) side. Here we assume $N_{tr} = 250$ trial times for the first step. The probability to generate entanglement for $N_{tr} = 250$ trial times is:

$$p_j = 1 - (1 - p_j^s)^{N_{tr}} \tag{16}$$

The probability distribution of the number of entangled pairs N_e between FPSA and Alice or Bob is:

$$\begin{aligned} p_N(N_e) &= \sum_{a_1,\ldots a_j} \frac{\prod_{i=1}^{N/2}(1-p_i)}{\prod_{i=1}^{j}(1-p_{a_i})} \prod_{i=1}^{j} p_{a_i} \\ &= \prod_{i=1}^{N/2}(1-p_i) \sum_{a_1,\ldots a_j} \prod_{i=1}^{j} q_{a_k} \end{aligned} \tag{17}$$

where $q_{a_k} = p_{a_k}/(1-p_{a_k})$. then the expectation number of qubit generated in this process with A and B can be expressed by:

$$\bar{N} = \sum_{N_e=1}^{N/2} \sum_{M=N_e+1}^{N/2} N_e p_N(N_e) p_N(M) + \sum_{N_e=1}^{N/2} N_e p_N(N_e)^2 \tag{18}$$

After $n = 250$ attempts, we need to redo the initialization. For one period, the total time cost in the first step is:

$$T_1 = t_{\rm cr} + n t_{\rm gate} \tag{19}$$

where $t_{\rm cr} = 40\ \mu s$ the CR state check time and $t_{\rm gate} = 5.5\ \mu s$ the gate time to prepare $|\alpha\rangle = \sqrt{\alpha}|0\rangle + \sqrt{1-\alpha}|1\rangle$.

Step 2: Local entanglement generation [9, 10]. As shown in main text, the probability to make the local

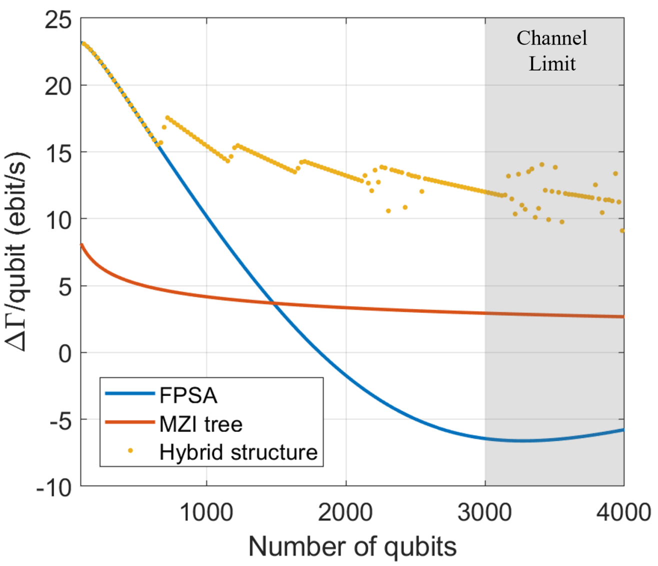

Supplementary Figure 5. Increase entanglement rate per added qubit for FPSA (blue) and MZI tree (red).

entanglement is $p_{\rm local} = (p_d\beta\exp(-t_{wg}N/2))^2/2$. The same dynamically tunable operations allow us to immediately attempt local entanglement as soon as distant entanglement is heralded, which decrease the total time needed for the second step from $T_2 = nt_{\rm localgate}/p_{\rm local}$ to $T_2 = t_{\rm localgate}/p_{\rm local}$, where $t_{\rm localgate} = 11\ \mu s$. Finally the rate Γ can be expressed as:

$$\Gamma = \frac{\bar{N}}{t_{\rm cr} + nt_{\rm gate} + t_{\rm localgate}/p_{\rm local}} \tag{20}$$

The simulation results are also checked through Monte-Carlo method.

B. Changed entanglement rate per added qubit

Another way to characterize the entanglement rate is the changed rate per added qubit, as shown in Supplementary Fig. 5. In the low number of qubits regime, add qubit to FPSA increase entanglement rate rapidly due to linear dependence of N. However, each additional qubit adds exponential loss. In the large number of qubits regime, the exponential loss dominate the decrease entanglement rate when $N > 1824$. The MZI tree shows increase entanglement rate in both regimes but shows a low increasing rate. The hybridization device of FPSA and MZI tree architectures shows large increase entanglement rate in all regimes, but the efficiency for adding qubits will still decrease in large number of qubits regime. The discontinuity are due to changed number of devices.

VII. SUPPLEMENTARY NOTE 7: STRAIN TUNING DESIGN

Supplementary Fig. 6(a) indicates the TE-like modes of the slow-light waveguide with the parameters shown in Table 1 in main text. By coupling the SiV transition at ν_0 to the slow-light region, we can thus funnel the coherent emission into waveguide modes near wave-vector $k_x(\nu_0)$, as shown in Supplementary Fig. 6(b).

The mean ZPL frequency $\Delta_{\rm ZPL}$ and ground state (excited state) orbital splittings Δ_{gs} (Δ_{es}) can be written as a function [3]:

$$\Delta_{\rm ZPL} = \Delta_{\rm ZPL,0} + (t_{\parallel,es} - t_{\parallel,gs})\epsilon_{zz} + (t_{\perp,es} - t_{\perp,gs})(\epsilon_{xx} + \epsilon_{yy}) \tag{21}$$

$$\Delta_{gs} = \sqrt{\lambda_{SO,gs}^2 + 4[d_{gs}(\epsilon_{xx} - \epsilon_{yy}) + f_{gs}\epsilon_{yz}]^2 + 4[-2d_{gs}\epsilon_{xy} + f_{gs}\epsilon_{zx}]^2} \tag{22}$$

$$\Delta_{es} = \sqrt{\lambda_{SO,es}^2 + 4[d_{es}(\epsilon_{xx} - \epsilon_{yy}) + f_{es}\epsilon_{yz}]^2 + 4[-2d_{es}\epsilon_{xy} + f_{es}\epsilon_{zx}]^2} \tag{23}$$

The strain terms and definitions are listed on Table S1. Considering the C transition of SiV, the optical shift caused by strain is shown in Eq.(19):

$$\Delta = \Delta_{{\rm ZPL},zz} + \sqrt{\lambda_{{\rm SO},gs}^2 + 4\Delta_{gs1}^2 + 4\Delta_{gs2}^2} - \lambda_{{\rm SO},gs} + \sqrt{\lambda_{{\rm SO},es}^2 + 4\Delta_{es1}^2 + 4\Delta_{es2}^2} - \lambda_{{\rm SO},es} \tag{24}$$

In principle, we need 5 degree of freedoms in the spectral addressing process. However, Δ_{es1} and Δ_{es2} (Δ_{gs1} and Δ_{gs2}) have similar structure and the tolerance of the spectral addressing is similar with the bandwidth of the frequency multiplexer (20 GHz) [11], so we only need 3 degree of freedoms to make spectral addressing process.

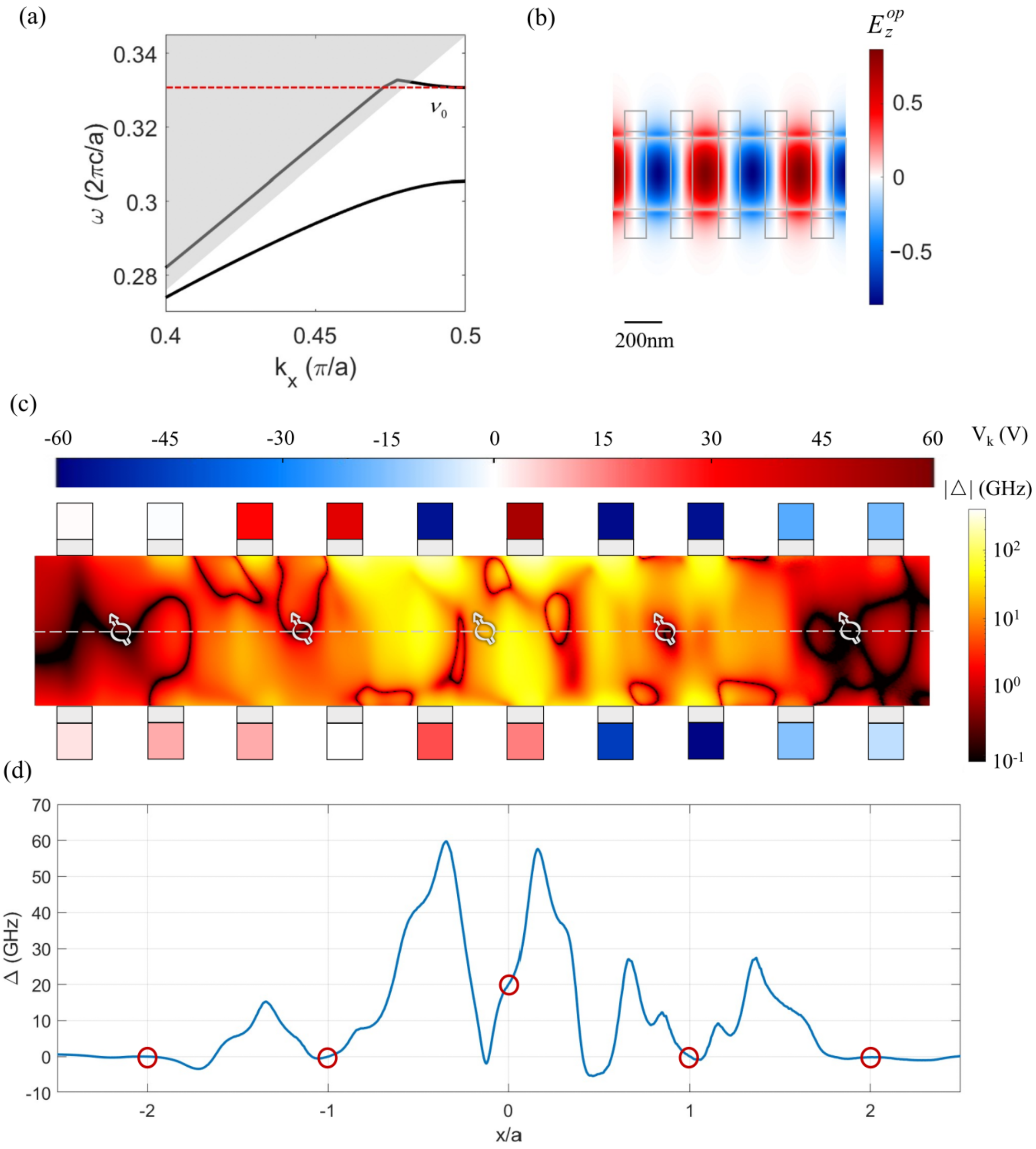

Supplementary Figure 6. (a). Photonic TE band structure of the FPSA slow-light waveguide using the parameters shown in Table 1. The shaded regions indicate the light cone for propagation in substrate. (b). The y-component of the electric field at the midplane of the diamond. (c). Optical transition tuning through strain tuning. Here we tune the second spin to have a 20 GHz strain tuning, while for all other spins to have less than 0.3 GHz strain tuning. (d). Total strain tuning frequency along the white dotted line in (a).

As shown in Supplementary Fig. 6(c), we have 4 electrodes (degree of freedoms) for a single spin. We show a spectral addressing where we set center spin in Supplementary Fig. 6(c) to have a 20 GHz strain tuning while all other spins have less than 0.3 GHz strain tuning, showing the feasibility to tune the optical transition based on the voltage settings.

[1] R. E. Evans, M. K. Bhaskar, D. D. Sukachev, C. T. Nguyen, A. Sipahigil, M. J. Burek, B. Machielse, G. H. Zhang, A. S. Zibrov, E. Bielejec, *et al.*, Photon-mediated interactions between quantum emitters in a diamond nanocavity, Science **362**, 662 (2018).

[2] L. Bernstein, A. Sludds, R. Hamerly, V. Sze, J. Emer, and D. Englund, Freely scalable and reconfigurable optical hardware for deep learning, Scientific reports **11**, 1

(2021).

[3] S. Meesala, Y.-I. Sohn, B. Pingault, L. Shao, H. A. Atikian, J. Holzgrafe, M. Gündoğan, C. Stavrakas, A. Sipahigil, C. Chia, *et al.*, Strain engineering of the silicon-vacancy center in diamond, Physical Review B **97**, 205444 (2018).

[4] J.-I. Matsuda, Measurements of leakage currents and the capacitance of the storage capacitor in a single dram cell, IEEE transactions on electron devices **41**, 391 (1994).

[5] C. Nguyen, D. Sukachev, M. Bhaskar, B. Machielse, D. Levonian, E. Knall, P. Stroganov, C. Chia, M. Burek, R. Riedinger, *et al.*, An integrated nanophotonic quantum register based on silicon-vacancy spins in diamond, Physical Review B **100**, 165428 (2019).

[6] Y. Chu, N. P. de Leon, B. J. Shields, B. Hausmann, R. Evans, E. Togan, M. J. Burek, M. Markham, A. Stacey, A. S. Zibrov, *et al.*, Coherent optical transitions in implanted nitrogen vacancy centers, Nano letters **14**, 1982 (2014).

[7] J.-H. Kim, S. Aghaeimeibodi, C. J. Richardson, R. P. Leavitt, and E. Waks, Super-radiant emission from quantum dots in a nanophotonic waveguide, Nano Letters **18**, 4734 (2018).

[8] P. C. Humphreys, N. Kalb, J. P. Morits, R. N. Schouten, R. F. Vermeulen, D. J. Twitchen, M. Markham, and R. Hanson, Deterministic delivery of remote entanglement on a quantum network, Nature **558**, 268 (2018).

[9] S. D. Barrett and P. Kok, Efficient high-fidelity quantum computation using matter qubits and linear optics, Physical Review A **71**, 060310 (2005).

[10] H. Bernien, B. Hensen, W. Pfaff, G. Koolstra, M. S. Blok, L. Robledo, T. Taminiau, M. Markham, D. J. Twitchen, L. Childress, *et al.*, Heralded entanglement between solid-state qubits separated by three metres, Nature **497**, 86 (2013).

[11] D. Seyringer, Design and simulation of 128-channel 10 ghz awg for ultra-dense wavelength division multiplexing, in *2012 14th International Conference on Transparent Optical Networks (ICTON)* (IEEE, 2012) pp. 1–4.

Supplementary Table I. Strain Parameters

Strain	Value
$t_{\parallel,es} - t_{\parallel,gs}$	-1.7 ± 0.1 PHz/strain
$t_{\perp,es} - t_{\perp,gs}$	0.078 ± 0.009 PHz/strain
d_{gs}	1.3 ± 0.1 PHz/strain
d_{es}	1.8 ± 0.2 PHz/strain
f_{gs}	-1.7 ± 0.1 PHz/strain
f_{es}	-3.4 ± 0.3 PHz/strain
Δ_{gs1}	$d_{gs}(\epsilon_{xx} - \epsilon_{yy}) + f_{gs}\epsilon_{yz}$
Δ_{gs2}	$-2d_{gs}\epsilon_{xy} + f_{gs}\epsilon_{zx}$
Δ_{es1}	$d_{es}(\epsilon_{xx} - \epsilon_{yy}) + f_{es}\epsilon_{yz}$
Δ_{es2}	$-2d_{es}\epsilon_{xy} + f_{es}\epsilon_{zx}$
$\Delta_{\mathrm{ZPL},zz}$	$(t_{\parallel,es} - t_{\parallel,gs})\epsilon_{zz}$
